# Managing Hypertension, Diabetes, and Cardiovascular Disease Risk via Short-Term Medical Trips: A Retrospective Longitudinal Study in Santo Domingo

**DOI:** 10.5334/aogh.3369

**Published:** 2022-01-13

**Authors:** Rose Baumann, Reuben Retnam, Carlos Mejia Hernandez, Victoria Edwards, Mark Ryan

**Affiliations:** 1 Department of General Internal Medicine, University of Minnesota Medical School, Minneapolis, MN, US; 2 Department of Internal Medicine, Virginia Commonwealth University, Richmond, VA, US; 3 Department of Biostatistics, Virginia Commonwealth University, Richmond, VA, US; 4 Department of Internal Medicine, TriHealth Good Samaritan Hospital, Cincinnati, OH, US; 5 Instituto Tecnológico de Santo Domingo (INTEC), Santo Domingo, Dominican Republic; 6 Howard University College of Medicine, Washington, DC, US; 7 Department of Family Medicine and Population Health, Virginia Commonwealth University, Richmond, VA, US

## Abstract

**Background:**

Short-term medical trips (STMTs) from high-resource countries frequently provide care in low and middle-income countries. Little existing literature objectively tracks the long-term outcomes of these interventions on the receiving populations over time to assess potential benefits and to ensure no harm is being done.

**Objectives:**

The purpose of this study was to objectively analyze the outcomes of a biannual STMT to Santo Domingo, Dominican Republic on hypertension (HTN), diabetes mellitus type 2 (DM2), and cardiovascular disease (CVD) risk over a five-year period (2015–2019).

**Methods:**

Data from 1655 patients was extracted from the electronic medical record. In patients who received treatment and had more than one visit, a linear mixed model was used to analyze effects on systolic blood pressure (SBP) and hemoglobin A1C (HbA1C) values over time. In patients with high CVD risk based on a non-laboratory-based assessment, provider compliance with prescribing an aspirin and statin was calculated and tracked over time.

**Results:**

In patients with HTN who received treatment, average SBP was 148.83 mmHg (SD = 23.96) at initial visit and demonstrated no change over time (Estimate: 0.68 mmHg/year increase, p = 0.46). HbA1C data was insufficient for analysis. Treatment for patients with high CVD risk with an aspirin and statin improved from 41.46% in 2015 to 70.51% in 2019.

**Conclusion:**

SBP in patients with HTN treated by this STMT demonstrated no significant change over time. Possible contributing factors included patient education, access and adherence to medications, and documentation of data. Provider compliance with appropriate prescribing was high for patients with HTN and DM2 and improved over time for patients with high CVD risk, serving as an indirect measure for potential long-term benefits on these populations. All STMTs should objectively track outcomes of their interventions to assess risks and benefits to the communities being served.

## Introduction

According to the World Health Organization (WHO), noncommunicable diseases (NCDs) currently account for 70% of deaths around the world [1] and similarly 72% of deaths in the Dominican Republic (DR) [2]. NCDs include cardiovascular disease (CVD), diabetes, chronic respiratory diseases, and cancers; contributing risk factors for these NCDs include hypertension (HTN), tobacco use, and obesity [3]. NCDs have become the leading causes of death in the DR [4]. According to the Global Burden of Disease Study, ischemic heart disease and stroke are the first and second primary causes of death in the DR, respectively, with diabetes as the fourth leading cause of death [4,5]. From 2009 to 2019, the percent of deaths by ischemic heart disease increased by 41.7% while deaths caused by diabetes increased by 43.6% [4].

Short-term medical trips (STMTs), typically groups of clinicians and non-clinician volunteers originating from high-resource settings, play a unique role in contributing to the care of NCDs in lower-resource settings. These trips may provide opportunities for access to education, screening for, and treatment of NCDs and their contributing factors in communities underserved by local medical providers and may provide a free source of medical care and medications for patients who may not otherwise be able to afford them [6]. However, STMT volunteers face the challenges of cultural, language, and financial barriers, as well as ensuring adequate follow-up after the group leaves [6]. The long-term effects of these STMT interventions on NCDs may be difficult to assess based on the episodic nature of these trips, and the literature is lacking in objective assessments of these projects’ outcomes.

In a systematic review of studies on STMTs, Sykes found that in 50 relevant studies, only 26% reported late patient outcomes, defined as an outcome beyond eight days after the intervention [7]. These late outcomes were all surgical, and none were medical [7]. Martiniuk et al. completed a review of literature on STMTs from 1985–2009, which found that 78% of published articles were descriptive rather than analytical [8]. Maki et al. developed extensive surveys to evaluate a medical mission, including questions on cost, efficiency, and sustainability to be answered by everyone involved including patients, hosts, and other personnel; these surveys were subjective [9]. Overall, the literature lacks objective analysis and evidence of long-term outcomes of STMT interventions.

However, understanding the long-term impact and ensuring no harm is done is ethically necessary. Volunteers must acknowledge the possibility that STMT presence may discourage patients from using a more consistent form of local medical care and may lead to an unhealthy reliance on an intermittent service for their medication supplies and healthcare needs. If this is occurring, presumably the desired outcomes of interventions would not be achieved. Furthermore, ensuring that providers are compliant with standard of care practices in their prescribing is ethically necessary for the long-term health outcomes of the patients being treated. Therefore, these outcomes must be measured and evaluated regularly. For the purpose of this paper, the authors define long-term impact as an outcome beyond the duration of the trip that can be objectively measured (rather than subjective), including factors that influence the long-term health of the individuals being treated. Specifically, this paper will focus on outcomes related to NCDs and their contributing factors. This paper will also evaluate STMT provider adherence to evidence-based guidelines for hypertension and type 2 diabetes mellitus (DM2) care, and United States Preventive Services Task Force (USPSTF) guideline-based preventive care for patients at high risk of CVD events.

The Dominican Aid Society of Virginia (DASV) has conducted semiannual week-long STMTs to the community of Paraiso just outside Santo Domingo, DR, since 2009. DASV STMT teams include physicians, medical students, pharmacists, pharmacy students, and other volunteers. Health profession students assist with triage, patient intake, and providing pharmacy services under the direct supervision of pharmacists, physicians, and healthcare providers. Other volunteers provide interpretation support in patient care settings. Licensed medical providers lead the medical encounters and are responsible for the plan of care determined with the patient. The STMT has maintained an electronic medical record (EMR) for all patients seen since 2015. Between 350 and 550 patients are seen during each trip and many patients are seen for follow-up visits on more than one return trip.

Diabetes mellitus type 2 (DM2), HTN, and CVD risk are NCDs that are managed with medications and patient education during these STMTs. Patients are generally provided with a 100-day supply of medications at no cost. All medications provided by the STMT are purchased locally to allow for familiarity in packaging and ensure all medications provided are readily available to the patients in the interim. Since 2017, patients with a history of DM2 are also evaluated using a point-of-care hemoglobin A1c (HbA1C) test. In addition, appropriate patients are assessed for CVD risk using a non-laboratory-based method which takes into account age, gender, diabetic status, body mass index (BMI), systolic blood pressure (SBP), and smoking status [10]. If CVD risk is determined to be high (>20–30% or >30% 5-year risk of MI [10]) low-dose aspirin and statin are prescribed to the patient by the STMT following evidence that these interventions reduce overall CVD risk, according to USPSTF guidelines in place at the time of this STMT [11,12].

Hochheimer et al. analyzed the long-term effects of interventions by this group on blood pressure between the years 2014 and 2016 (using previously available paper documentation from these years) [13]. The study found a significant decrease in diastolic blood pressure in returning patients who attended both the 2014 and 2016 clinics, compared to an internal comparison group of age- and gender- matched patients who only attended the clinic in either 2014 or 2016. The purpose of this current study is to take these findings a step further, using a more comprehensive data set available in the EMR with numerous patient visits over time (2015–2019) to evaluate effects of this STMT on SBP, an NCD contributing factor, and DM2, an NCD. This current study will also evaluate provider compliance to standard of care in prescribing for patients at high risk of CVD events, with CVDs, including coronary heart disease and cerebrovascular disease, being NCDs. Desired outcomes for patient benefit include a decrease in SBP over time, a decrease in HbA1C over time, and appropriate prescribing for those with high CVD risk in order to decrease long-term risk of CVD events.

The purpose of this study is to provide objective assessment of interventions by this STMT on the following three aspects of NCDs in the DR: HTN, DM2, and CVD risk. This purpose will be accomplished by answering the following three questions:

1. In adult patients with HTN who attended this STMT clinic more than once between 2015 and 2019, did prescribing of an antihypertensive medication by the STMT team lead to a significant change in SBP over time?
2. In adult patients with DM2 who attended this STMT clinic more than once between 2015 and 2019, did prescribing of an antidiabetic medication by the STMT team lead to a significant change in HbA1C value over time?
3. In adult patients with high CVD risk based on the non-laboratory-based risk assessment, what percentage of patients seen by STMT providers were appropriately prescribed a low-dose aspirin and statin medication and how did this percentage change over time?

## Methods

### Patient Selection

For questions 1 and 2, subjects were adult patients who attended the STMT clinic more than once between 2015 and 2019 and had necessary data available in the EMR from more than one visit. For the HTN analysis (question 1), the patient must have had documentation of an SBP of >140 mmHg, a listed diagnosis of HTN, previous prescription of an antihypertensive, or a combination of these criteria; all patients must have had documentation of an SBP at two separate visits at least six months apart. For the DM2 analysis (question 2), patients must have had a documented HbA1C of >6.5% mmol/mol, a documented diagnosis of DM2, previous prescription of an antidiabetic medication, or a combination of these criteria; all patients must have had at least two HbA1C values documented from two separate visits at least six months apart. For both the HTN and DM2 analyses, the patient must have been appropriately prescribed an antihypertensive or antidiabetic medication, respectively, at a visit that had a subsequent visit for comparison at least six months later.

For question 3, patients must have been adults who had a single STMT clinic visit with enough data to determine CVD risk based on non-laboratory-based assessment method [9] or documentation of low, moderate, or high CVD risk in the chart by a provider based on this assessment. Necessary data for assessment included age, gender, height and weight (to calculate BMI), SBP, diabetic status, and smoking status. The patient encounter must also have included documentation of the presence or absence (which was assumed if no documentation) of a prescription for a low-dose aspirin and statin.

Due to the effect of certain acute illnesses on blood pressure, patient visits with the following acute illnesses were excluded from the analysis: sinusitis, pneumonia, bronchitis, fever, influenza, sickle cell crisis, acute back pain, asthma or chronic obstructive pulmonary disease exacerbation, or cellulitis. Patients were determined to meet exclusion criteria after clinician assessment of the medical record.

### IRB Approval

Institutional Review Board (IRB) approval (case #HM20015895) was obtained through Virginia Commonwealth University. Due to retrospective analysis, informed consent was not obtained.

### Statistical Analysis

Data from the EMR (Team fEMR *https://teamfemr.org/*) was extracted into a Microsoft Excel spreadsheet. A reviewer evaluated each patient encounter and reformatted the data into organized columns suitable for the analysis. Appropriate summary statistics were computed for each variable. Missing values were not considered in the calculations of summary statistics.

Analysis for the first two research questions was performed utilizing linear mixed models with time since first visit, age, and gender as covariates. The outcomes, respectively, were mean SBP and HbA1C values. Irregular observation times were handled via autoregressive correlation structures.

Analysis for the third research question was performed via identification of patients with high CVD risk via the NHANES 1 non-laboratory-based assessment method [10]. The proportion of high-risk patients prescribed appropriate therapy (aspirin plus statin) was computed and reported. Since the question pertains to this specific STMT, no inferential statistics were reported for this question. Percent of compliance with prescription of the appropriate therapy per year over time by the STMT providers was also evaluated.

Statistical significance was evaluated at the 0.05 level. All analyses were performed in R 3.5.2.

## Results

### Patient Characteristics

Data from 1655 patients was extracted from the EMR. Of the 1655 patients with recorded encounters, 31 patients met exclusion criteria, leaving 1624 patients included in the analyses and statistics below. At their first visit, the patients’ mean age was 47.92 years (SD = 16.62 years) (***Table 1***). Most patients were female (73.88%, ***Table 1***). Patients’ first SBPs averaged 134.56 mmHg (SD = 24.79 mmHg, ***Table 1***), while HbA1c levels averaged 7.24 mmol/mol (SD = 2.2 mmol/mol, ***Table 1***). Of the 1624 patients, 529 patients had more than one visit (32.57%). Specifically, 273 (16.81%) patients had two visits, 103 (6.34%) had three visits, 58 (3.57%) had four visits, 41 (2.52%) had five visits, 26 (1.60%) had six visits, 18 (1.11%) had seven visits, 8 (0.49%) had eight visits, and 2 (0.12%) had nine visits. Patients had an average of 1.66 visits (SD = 1.34).

**Table 1:** Summary Statistics of Patients at First Visit.


VARIABLE	SUMMARY MEAN (SD) OR N (%)

Age (yrs)	47.92 (16.62)

Gender (Male)	401 (26.12%)

Smoking	101 (6.22%)

SBP (mmHg)	134.56 (24.79)

HgbA1C (mmol/mol)	7.24 (2.20)

Diagnosis of HTN	849 (52.31%)

Diagnosis of DM2	247 (15.21%)


### Question 1: Hypertension Analysis Results

At the first visit, 849 (52.31%) patients had essential HTN and 735 of these (86.57%) were prescribed an antihypertensive medication. The mean SBP in patients with HTN at first visit was 148.83 mmHg (SD = 23.96). When separated by visit number, 86.57–100% of patients with HTN were appropriately prescribed an antihypertensive medication by the STMT (***Table 2***). Medications prescribed included hydrochlorothiazide, atenolol, amlodipine, lisinopril, and losartan. Among patients prescribed a medication at first visit, 276 (37.55%) had follow-up visits between six months and three years later and had SBP data on those visits. Patients on antihypertensive medications, on average (controlling for gender and age), did not experience change in mean SBP over time (estimate: 0.68 mmHg/year increase, p = 0.46, ***Table 3***). Males on antihypertensives had a higher blood pressure by 8.66 mmHg compared to females (p = <0.01, ***Table 3***). Higher age was associated with slightly higher SBP by 0.23 (p = 0.03, ***Table 3***).

**Table 2:** Frequencies and Proportions of Patients with Hypertension/Diabetes and on Appropriate Therapy Per Visit.


VISIT	HYPERTENSIVE	HTN ON APPROPRIATE THERAPY	DIABETIC	DM2 ON APPROPRIATE THERAPY

1	849 (52.31%)	735 (86.57%)	247 (15.21%)	214 (86.64%)

2	347 (65.60%)	308 (88.76%)	132 (24.95%)	104 (78.79%)

3	194 (75.78%)	175 (90.21%)	74 (28.91%)	63 (85.14%)

4	126 (82.35%)	122 (96.83%)	60 (39.22%)	48 (80.00%)

5	80 (84.21%)	78 (97.50%)	45 (47.37%)	43 (95.56%)

6	49 (90.74%)	48 (97.96%)	22 (40.74%)	20 (90.91%)

7	26 (92.86%)	26 (100%)	14 (50%)	13 (92.86%)

8	10 (100%)	9 (90%)	8 (80%)	7 (87.50%)

9	2 (100%)	2 (100%)	2 (100%)	2 (100%)


**Table 3:** Linear Mixed Model Results for Systolic Blood Pressure in Patients on Antihypertensive Medications.


PREDICTORS	ESTIMATES (mmHg)	95% CI	p VALUE

Time (years)	0.68	–1.15–2.51	0.46

Male – Female	8.66	3.37–13.95	**<0.01**

Age (years)	0.23	0.02–0.44	**0.03**


### Question 2: Diabetes Analysis Results

In total, 247 (15.21%) of patients had DM2 (***Table 1***) and 214 of these (88.64%) were prescribed an antidiabetic medication. At first visit, HbA1c levels averaged 7.24 mmol/mol (SD = 2.2 mmol/mol, ***Table 1***). When separated by visit number, 86.64–100% of patients with DM2 were prescribed an antidiabetic medication by the STMT (***Table 2***). Medications prescribed included metformin and glyburide. Among patients prescribed a medication, 116 (54.21%) had follow-up visits between 6 months and 1 year later; however, only 18 patients (15.51%) had HbA1C data at these follow-up visits. Therefore, not enough data existed to properly assess whether or not prescription of antidiabetic medication led to a change in HbA1C values over time.

### Question 3: CVD Risk Results

At first visit, 25.06% of patients had high CVD risk. Compliance with prescription of an aspirin and statin by the STMT to patients with high CVD risk increased over time. In 2015, 41% (N = 34) of patient encounters with high CVD risk (N = 82) were prescribed an aspirin and a statin; by 2019, this number had increased to 70.51% (N = 110) of those with high CVD risk (N = 156) (***Table 4***). The statin prescribed was simvastatin in nearly all instances.

**Table 4:** Prescriber Compliance Proportions of Prescription of Aspirin + Statin Over Time for Patients with High Cardiovascular Disease Risk.


COMPLIANCE OF PRESCRIPTION	2015	2016	2017	2018	2019

**Noncompliant**	48 (58.54%)	79 (47.02%)	79 (37.26%)	109 (41.60%)	46 (29.49%)

**Compliant**	34 (41.46%)	89 (52.98%)	133 (62.74%)	153 (58.40%)	110 (70.51%)


## Discussion

STMTs are common and often address and treat NCDs including DM2, HTN, and CVD risk in low and middle-income countries. In 2008, Maki et al. estimated greater than 6000 STMTs from the US per year [9], and this number has likely only increased. However, little objective data regarding the long-term effects of these trips exists in the literature. Overall, this study successfully analyzed data from a biannual STMT to Santo Domingo, DR from over 1600 patients including multiple encounters over several years.

Most patients with HTN (86–100%, ***Table 2***) were appropriately prescribed antihypertensive medications by the STMT; in other words, patients with elevated SBP had pharmacotherapy initiated or escalated after evaluation by a medical provider. Over the course of four years, patients with HTN did not experience a significant change in SBP over time (estimate: 0.68 mmHg/year increase, p = 0.46, ***Table 3***). With a statistically insignificant p value, these results are challenging to interpret. As blood pressure is known to increase with increasing age [14], and considering that in this study older age was associated with higher blood pressures among STMT patients, the authors are reassured that SBP remained stable over time and did not increase significantly. However, data was not able to demonstrate a significant decrease in SBP over time, as would be desired with appropriate therapy.

Many factors may contribute to this lack of desired decrease in SBP over time. Proper patient education by the team on how to take the medications is important to obtaining results. However, in a previous study on this same population, patients surveyed with a post-visit “medication quiz” averaged 91% accuracy in identifying the indication each medication was prescribed for [15]. Although measures of educational efforts from STMTs may not be sufficient to fully assess long-term adherence or patients’ ability to change chronic health behaviors, this data suggests that one aspect of education is adequate and less likely to be contributing to the results in this study. Of note, the quiz did not assess if patients understood how to take the medication or the importance of taking it daily, which are other potentially contributing factors. For this STMT, clinical documentation also did not specify the nature of patient-oriented guidance regarding therapeutic lifestyle changes and self-management, long-term medication adherence, or patient motivation and readiness for change.

Access to medications is more likely playing a role. In surveying patients receiving care by this STMT, Castro et al. found that although 63% of patients had insurance, 83% reported that insurance never or rarely paid for medications [6]. This STMT provides 100-days’ worth of medications but returns every six months; patients must obtain medications on their own locally in the interim. If this practice is not happening due to access and patients are running out of medication, this issue may explain the reason blood pressure is not decreased at a visit six months or more later. Adherence to medication, particularly on the day of the visit, may impact the blood pressure measurement that was taken that day and included in the analysis. While age and gender were accounted for in the analysis, other factors affecting blood pressure such as weight changes, diet changes, and other underlying conditions were not evaluated in this study. Other factors which might affect blood pressure results include such issues as wait time, environmental conditions, and distance of travel on the day of evaluation.

Most patients with DM2 (86–100%, ***Table 2***) had pharmacotherapy initiated or escalated by a licensed medical professional in the context of their medical encounter. However, HbA1C data was insufficient in the EMR to determine the effects on this value over time. Point-of-care HbA1C machines are a new addition to the STMT clinical setting since late 2017. As no clear place to document the lab value in the EMR exists, some tests were likely acquired but not recorded in a retrievable location.

The results of this study highlight high compliance on the part of STMT providers with appropriate prescribing over time. As noted above, patients with HTN and DM2 were prescribed appropriate medications for their diagnosis the majority of the time by the STMT; the high percent of appropriate prescribing remained stable when compared over the number of visits (***Table 2***). Furthermore, patients with high CVD risk were appropriately prescribed an aspirin and statin 70.51% of the time by 2019, a progressive increase from 41.46% of the time in 2015 (***Table 4***). In the context of different volunteers attending the STMT every six months, these results reveal proper orientation to providers at the start of the trip is likely happening, and that clinical data and clinical decision support is being provided appropriately to the prescribers joining the STMT. Treatments for hypertension and type 2 diabetes were in-line with evidence-based practice recommendations in the context of local medication costs and access. Medications provided for CVD prevention were in accordance with USPSTF guidelines in place at that time [11,12] and were known to decrease morbidity in patients with high CVD risk.

Assessing true patient-oriented outcomes (for example, prevention of myocardial infarction, prevention of complications from type 2 diabetes) should be the gold standard by which NCD treatments and interventions are assessed. However, these outcomes require long-term tracking of data and patient outcomes, which are complicated by the nature of STMTs. As a shorter-term outcome, evaluating objective evidence of provider adherence to evidence-based care which have been demonstrated to reduce patient risks of illness and disease may be important measures of the function of STMTs in managing NCDs.

Limitations in the data should be acknowledged. Documentation in the EMR was incomplete at times, leading to unintentionally excluded encounters. Also, some patients may have more than one record in the EMR, which limits the ability to evaluate patients’ outcomes over time. These multiple records may exist for several reasons, including patients’ lack of government-issued identifications, phonetic spellings of patients’ names, and incorrectly recorded dates of birth. In the HTN analysis, medication name and dose were not included in the analysis, which could have played a role in the examined results. The majority of patients with HTN (62.45%) did not return to the STMT for follow-up; data cannot determine if these patients were lost to follow-up of medical care or if they instead sought care from a local provider. Lack of these patients’ follow-up with the STMT may overestimate or underestimate the change in SBP. Finally, the data collected could not measure or assess how providers address therapeutic lifestyle changes in the context of the medical visits.

While the results show potentially positive effects of this STMT (improvement in evidence-based prescribing, identifying, and providing care to high-risk patients), they also show some areas for further development for improvement in patient care and outcomes. This STMT should consider the cost and logistics of providing six-months’ worth of medications to patients to bridge gaps between trips during which time access to medications may be a concern. Alternatively, a more sustainable model would involve a collaboration with local providers who could evaluate patients in the interim and assist with access to local medications. Dominican providers, translators, and volunteers who participate in the STMT could potentially play a vital role in coordinating this care. Unfortunately, past efforts to link patients to the local government health centers have not been successful. Reasons for this are unclear, but from patient reports and authors’ experiences, factors include lack of patient health insurance, lack of legal documentation required to access health care, the physical challenges patients experience in accessing local medical care, and the lack of funding experienced in public health primary care clinics. In the future, emphasis on improved documentation could allow for better assessment of long-term effects of this trip. Future research can incorporate means such as surveys to assess the effects of life-style factors on SBP and DM2, as discussed above. Further assessment of and improvement in patient education may also impact future outcomes.

STMTs which take an active role in providing care for NCDs and conditions which contribute to NCDs should also be expected to assess the outcomes of the care they provide. Which outcomes should be measured, and at what frequency, are unclear, but they could reflect quality measures commonly used in clinical practice in the United States (for example, blood pressure control, diabetes control as determined by A1c levels). However, obtaining and tracking these measurements are complicated by inconsistent patient attendance at any given STMT, challenges with medical record access and storage, and the long timeline needed to evaluate for some important patient-oriented outcomes. The fact that many of the clinical guidelines used in medical practice in the United States are established for use in formal medical settings (for example, blood pressure measurements following specific parameters) may also complicate their use in outreach clinics which might be set up in repurposed schools, community buildings, or sheltered outdoor areas.

Given the significant investment in cost and resources for each STMT and the increasing numbers of STMTs which travel each year from the United States to low and middle-income countries, the long-term impact of STMTs is a necessary part of each project’s outcomes. Given that patients may come to depend on STMTs to provide at least a portion of their medical care and the risk that STMTs may disrupt local patterns of healthcare access, the authors encourage all STMTs to begin a process of tracking and assessing outcomes of patients who seek care from STMTs to ensure that no harm is being done as well as to assess for desired benefits. The model of this study can be reproduced by other STMTs to objectively analyze the effects of their interventions.

## Conclusions

STMTs to low and middle-income countries are common but their long-term effects are rarely objectively measured. This study provides an example of objective evaluation of the effects of a biannual STMT to the DR on HTN, DM2, and CVD risk over a five-year period. Results showed no significant change in SBP over time and insufficient data to analyze HbA1C. Overall, providers on this trip demonstrated high compliance with appropriate prescribing for HTN and DM2 and improved prescribing of aspirin and statin for high CVD risk over time. Future efforts of this STMT should focus on improved documentation and ensuring continuity of patient data, consideration of providing a larger supply of medications, and improved collaboration with local providers. All STMTs should consider using similar models to track outcomes to assess benefits and ensure that no harm is being done to patients who seek care from STMTs.

## References

[1] World Health Organization. Major NCDs and their risk factors. Published April 7, 2016. Accessed September 29, 2020. https://www.who.int/ncds/introduction/en/.

[2] Dominican Republic. World Health Organization – Noncommunicable Diseases (NCD) Country Profiles, 2018. Published 2018. Accessed February 6, 2020. https://www.who.int/nmh/countries/dom_en.pdf.

[3] World Health Organization. Noncommunicable diseases. Published June 1, 2018. Accessed September 29, 2020. https://www.who.int/en/news-room/fact-sheets/detail/noncommunicable-diseases.

[4] Dominican Republic. Institute for Health Metrics and Evaluation. Published September 18, 2017. Accessed March 19, 2021. http://www.healthdata.org/dominican-republic.

[5] Global Burden of Disease (GBD) 2017 study findings. Institute for Health Metrics and Evaluation. Published April 3, 2019. Accessed September 29, 2020. http://www.healthdata.org/presentation/global-burden-disease-gbd-2017-study-findings.

[6] Castro B, Ing L, Park Y, Abrams J, Ryan M. Addressing noncommunicable disease in Dominican Republic: barriers to hypertension and diabetes care. Ann Glob Health. 2018; 84(4): 625–629. DOI: 10.29024/aogh.2370 30779509 PMC6748242

[7] Sykes KJ. Short-term medical service trips: A systematic review of the evidence. Am J Public Health. 2014; 104(7): e38–e48. DOI: 10.2105/AJPH.2014.301983 PMC4056244 24832401

[8] Martiniuk AL, Manouchehrian M, Negin JA, Zwi AB. Brain gains: A literature review of medical missions to low and middle-income countries. BMC Health Serv Res. 2012; 12: 134. DOI: 10.1186/1472-6963-12-134 22643123 PMC3474169

[9] Maki J, Qualls M, White B, Kleefield S, Crone R. Health impact assessment and short-term medical missions: A methods study to evaluate quality of care. BMC Health Serv Res. 2008; 8: 121. DOI: 10.1186/1472-6963-8-121 18518997 PMC2464597

[10] Gaziano TA, Young CR, Fitzmaurice G, Atwood S, Gaziano JM. Laboratory-based versus non-laboratory-based method for assessment of cardiovascular disease risk: The NHANES I follow-up study cohort. Lancet. 2008; 371(9616): 923–931. DOI: 10.1016/S0140-6736(08)60418-3 18342687 PMC2864150

[11] Aspirin for the prevention of cardiovascular disease: Preventive medication. United States Preventive Services Task Force. Published March 15, 2009. Accessed November 30, 2021. https://www.uspreventiveservicestaskforce.org/uspstf/recommendation/aspirin-for-the-prevention-of-cardiovascular-disease-preventive-medication.

[12] Statin use for the primary prevention of cardiovascular disease in adults: Preventive medication. United States Preventive Services Task Force. Published November 13, 2016. Accessed November 30, 2021. https://www.uspreventiveservicestaskforce.org/uspstf/recommendation/statin-use-in-adults-preventive-medication.

[13] Hochheimer C, Khalid M, Vy M, Chang G, Tu D, Ryan M. Addressing noncommunicable disease on short-term medical trips: A longitudinal study of hypertension treatment in Santo Domingo. Ann Glob Health. 2017; 83(3–4): 471–477. DOI: 10.1016/j.aogh.2017.10.004 29221519

[14] Pinto E. Blood pressure and ageing. Postgrad Med J. 2007; 83(976): 109–114. DOI: 10.1136/pgmj.2006.048371 17308214 PMC2805932

[15] Morales D, Clay W, Khamishon R, et al. Bridging the gap: Including patient voices in short-term medical mission evaluations. Ann Glob Health. 2019; 85(1): 86. DOI: 10.5334/aogh.2431 31225961 PMC6634370

